# Clinical advances and challenges of anti-angiogenic targeted therapy in gastric cancer

**DOI:** 10.3389/fonc.2025.1654300

**Published:** 2025-10-23

**Authors:** Zhongyue Wu, Jiaojiao Ni, Qi Xu

**Affiliations:** ^1^ Department of Hepato-Pancreato-Biliary & Gastric Medical Oncology, Zhejiang Cancer Hospital, Hangzhou Institute of Medicine (HIM), Chinese Academy of Sciences, Hangzhou, China; ^2^ Postgraduate Training Base Alliance of Wenzhou Medical University (Zhejiang Cancer Hospital), Hangzhou, Zhejiang, China; ^3^ Zhejiang Key Laboratory of Prevention, Diagnosis and Therapy for Gastrointestinal Cancer, Hangzhou, China

**Keywords:** gastric cancer, anti-angiogenic, VEGFR inhibitors, biomarkers, clinical trials, precision oncology

## Abstract

Gastric cancer (GC) is a malignant neoplasm with one of the highest incidence and mortality rates. However, therapeutic options remain limited for advanced disease. Angiogenesis, a fundamental process in tumor progression, has emerged as a key therapeutic target. To comprehensively evaluate the clinical advancements of anti-angiogenic targeted drugs, this review conducted a systematic search and collation of pertinent literature, While, only ramucirumab achieved regulatory approval for second-line therapy, emerging agents including apatinib and fruquintinib demonstrate significant clinical benefits, particularly in combination with immunotherapy. The review also classifies and summarizes biomarkers with potential predictive value for treatment response, and discusses the current major challenges and potential optimization strategies. This analysis identifies significant gaps in predictive biomarkers and emphasizes that patient stratification and rational combination strategies are essential for optimizing therapeutic outcomes.

## 1 Background and mechanisms of anti-angiogenic therapy

Based on the latest global cancer statistics, new cases of GC and cancer-related deaths reached 960,000 and 650,000 in 2022. This places GC in the fifth position globally for both new incidence and cancer-related mortality (1). Notably, the global incidence of GC exhibits pronounced geographical disparities. The highest incidence rates are concentrated in East Asia and Eastern Europe, whereas regions such as Northern Europe, North America, and Africa report relatively lower incidences. Regarding treatment approaches, systemic chemotherapy has shown some survival benefit in advanced GC patients. However, the median overall survival (mOS) for those receiving conventional chemotherapy remains stagnant at approximately 12 months. In recent years, with the progressive advancement of personalized precision medicine, novel therapeutic modalities, including targeted therapy and immunotherapy, have increasingly become focal points of international research. Among these emerging strategies, anti-angiogenic targeted therapy has emerged as a highly promising approach, demonstrating substantial clinical utility in the management of GC.

Angiogenesis is a pivotal biological process in the development and metastasis of solid tumors. It functions as the principal pathway for delivering essential nutrients and oxygen to tumor tissues (2). In this complex process, vascular endothelial growth factor (VEGF), secreted by both tumor cells and stromal cells, serves as a key regulator of both physiological and pathological angiogenesis (3). The VEGF family comprises five distinct ligands, including VEGF-A, VEGF-B, VEGF-C, VEGF-D, and placental growth factor (PLGF), along with three receptor subtypes: VEGFR-1, VEGFR-2, and VEGFR-3 (4). When VEGF ligands bind to their corresponding VEGFRs on endothelial cell surfaces, elaborate signaling cascades are triggered. These cascades activate tyrosine kinase (TK) domains in the cytoplasmic regions of the receptors. The activation of TK promotes endothelial cell proliferation, migration, and resistance to apoptosis, ultimately contributing to accelerated tumor growth and metastatic spread (5).

**Figure 1:**
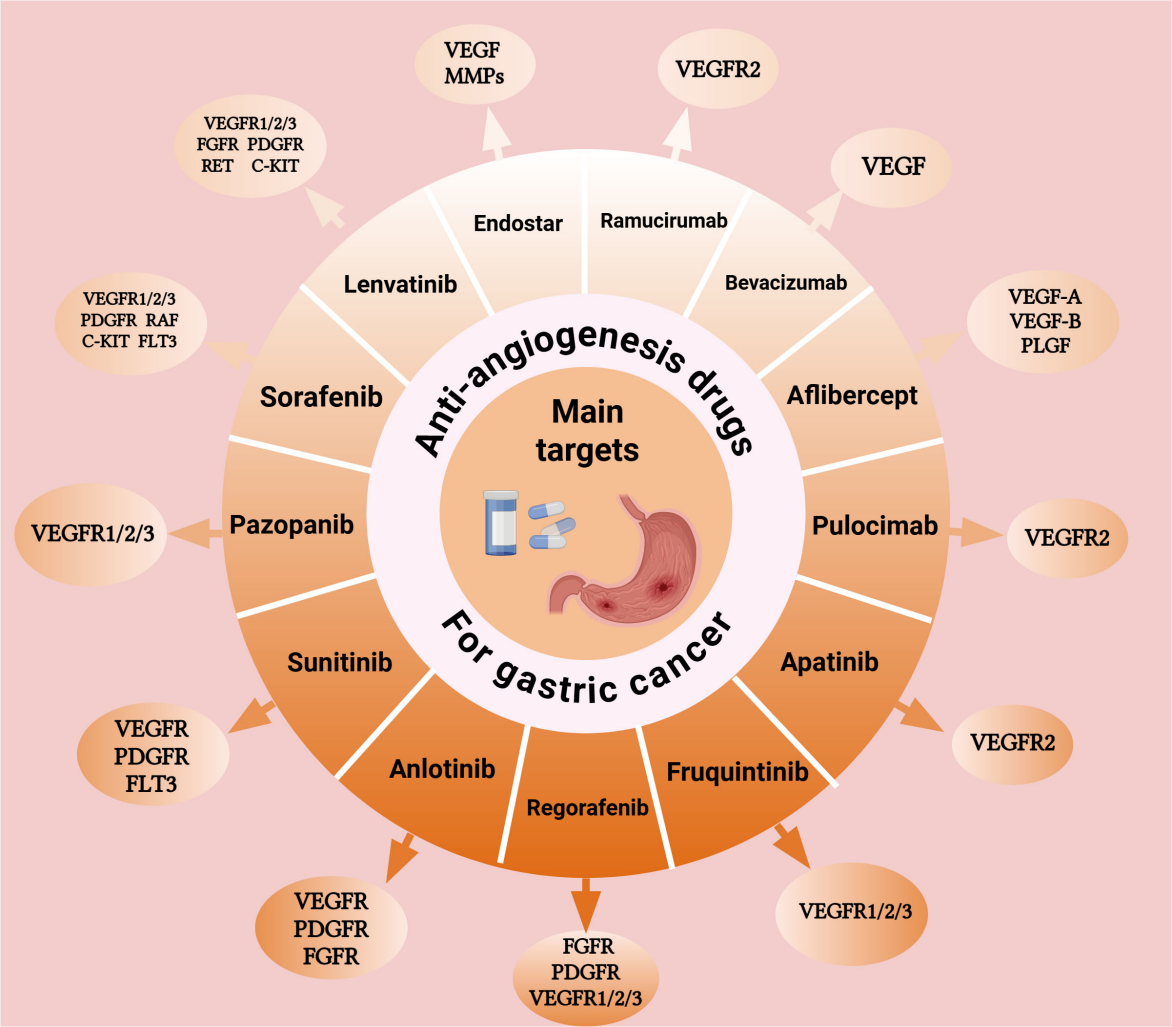
Mechanisms of action and molecular targets of anti-angiogenic drugs in gastric cancer Schematic illustration of the VEGF signaling pathway and target sites of anti-angiogenic agents in gastric cancer treatment. Monoclonal antibodies target VEGF ligands or receptors, while small-molecule tyrosine kinase inhibitors block multiple angiogenic pathways. These agents inhibit endothelial cell proliferation and migration, ultimately suppressing tumor angiogenesis. Abbreviations: VEGF, vascular endothelial growth factor; VEGFR, VEGF receptor. Created with BioRender.com. (Agreement number: ID28T9B5HK).

Over the past decade, anti-angiogenic targeted therapies have achieved remarkable progress in the treatment of advanced GC. Following the introduction of ramucirumab in 2014, a series of tyrosine kinase inhibitors (TKI) have emerged, thereby paving the way for a new therapeutic approach in the management of advanced GC. Anti-angiogenic drugs can be primarily classified into three major groups: (1) Monoclonal antibodies targeting VEGF or VEGFR, of which bevacizumab and ramucirumab are prominent representatives. In addition, novel bispecific antibodies, such as ivonescimab that simultaneously targets programmed cell death protein-1 (PD-1) and VEGF, have become an important subgroup; (2) Small-molecule TKIs, including apatinib and fruquintinib as representative examples; (3) Recombinant human vascular endothelial inhibitors, like endostar, which inhibit angiogenesis via multi-target mechanisms. In this review, we systematically elaborate on the mechanisms and targets of these anti-angiogenic drugs (**Figure 1**). The figure illustrates representative drugs of three major drug categories and their different targets in the angiogenic pathway. Then, we comprehensively summarize the advancements in their clinical trials and biomarker research, and discuss the major challenges and potential solutions.

While foundational reviews have established the theoretical framework for anti-angiogenic therapy in gastric cancer, significant gaps persist in the rapidly evolving clinical landscape (6, 7). Recent landmark trials including FRUTIGA and DRAGON IV have fundamentally altered treatment paradigms, while emerging therapeutic modalities such as bispecific antibodies and recombinant inhibitors extend beyond traditional VEGF blockade strategies. Furthermore, the field lacks systematic integration of predictive biomarkers across circulating, tissue-based, and multi-omics platforms with explicit clinical validation status. This review addresses these critical knowledge gaps by synthesizing the most recent clinical developments, novel therapeutic approaches, and advances in precision medicine to provide an updated framework for anti-angiogenic therapy in gastric cancer management.

## 2 Classification and clinical application of anti-angiogenic drugs in gastric cancer

### 2.1 Monoclonal antibody

#### 2.1.1 Ramucirumab

Ramucirumab stands as a significant breakthrough in GC therapy, being the first anti-angiogenic monoclonal antibody approved by the US Food and Drug Administration (FDA) for the second-line treatment of advanced GC. As a fully humanized immunoglobulin G1 (IgG1) monoclonal antibody, it inhibits VEGF ligand-induced endothelial cell proliferation and migration. It achieves this by specifically binding to VEGFR-2 and blocking the binding of VEGF-A, VEGF-C and VEGF-D. This mode of action results in the obstruction of neovascularization, thereby restricting the supply of nutrients to tumor tissue and ultimately inducing tumor cell death (8, 9). The outcomes of prior clinical trials involving ramucirumab are presented in **Table 1**, summarizing efficacy and safety data across different treatment lines.

**Table 1:** Summary of ramucirumab clinical trials in gastric cancer across multiple lines of therapy.

Treatment line	Phase	Registration number	Drugs (number)	ORR	DCR	mPFS (months) or PFS rate	mOS (months) or OS rate	Grade3/4 adverse events	References
2L	III	NCT00917384/REGARD	Ramucirumab(238)	3.3%	48.7%	2.1	5.2	57.00%	(10)
			Placebo(117)	2.6%	23.1%	1.3	3.8	58.00%	
	III	NCT01170663/RAINBOW	Ramucirumab+Paclitaxel(330)	27.90%	80.00%	4.4	9.6	/	(11)
			Placebo+Paclitaxel(335)	16.10%	63.60%	2.9	7.4	/	
	III	NCT02898077/RAINBOW-Asia	Ramucirumab+Paclitaxel(294)	26.50%	76.90%	4.14	8.71	76.00%	(12)
			Placebo+Paclitaxel(146)	21.90%	72.60%	3.15	7.9	66.00%	
	II	jRCTs011180029/HGCSG1603	Ramucirumab+Irinotecan(35)	25.90%	85.20%	4.2	9.6	>10%	(13)
	II	jRCTs031180061/JACCROGC-09)	Paclitaxel+Ramucirumab(47)	25%	75.00%	4.7	/	83.00%	(14)
	II	UMIN:000023515/YCOG1601	Ramucirumab+Paclitaxel(25)	/	88.00%	6.87	13.43	84.00%	(15)
	II	NCT03081143	FOLFIRI+Ramucirumab(72)	22%	/	6.8	/	75.00%	(16)
			Paclitaxel+Ramucirumab(38)	11%	/	7.6	/	68.00%	
	II	NCT01983878	Ramucirumab(36)	0%	30.60%	6.6	8.6	52.80%	(17)
	I/II	UMIN000025947	Nivolumab+Paclitaxel+Ramucirumab(43)	37.20%	83.70%	46.5% (6months)	13.1	32.60%	(18)
	I	NCT03193918	Crenolanib+Paclitaxel+Ramucirumab(14)	42%	/	43% (6months)	/	≥19%	(19)
	Review	/	Ramucirumab+Paclitaxel(20)	10.00%	55.00%	2.7	6.3	45.00%	(20)
	Review	/	FOLFIRI+Ramucirumab(29)	23.00%	79.00%	6	13.4	6.90%	(21)
1L	III	NCT02314117/RAINFALL	Ramucirumab+Cisplatin+5-FU(326)	41.10%	81.90%	5.72	11.2	/	(22)
			Placebo+Cisplatin+5-FU(319)	36.40%	76.50%	5.39	10.7	/	
	II	NCT02539225/RAINSTORM	S-1/Oxaliplatin+Ramucirumab(96)	58.20%	91.00%	6.34	14.65	29.20%	(23)
			S-1/Oxaliplatin+Placebo(93)	50.00%	87.00%	6.74	14.26	23.70%	
	II	jRCTs071180066/KSCC1701	S-1+Ramucirumab(48)	41.9%	/	5.8	16.4	≥15%	(24)
2L and 3L	II	UMIN:000022956	2L:Ramucirumab+Paclitaxel(11)	/	/	/	13.1	/	(25)
			3L:Ramucirumab+Irinotecan(5)	/	/	3.98	9.99	/	
	II	JapicCTI-194596	2L:TAS102+Ramucirumab(33)	9.00%	85%	5.9	/	84.00%	(26)
			3L:TAS102+Ramucirumab(31)	16.00%	77.00%	5.3	/	81.00%	
≥3L	Review	/	TAS102+Ramucirumab(36)	25.80%	58.10%	2.9	7.9	72.2%	(27)
			TAS102(70)	5.00%	38.30%	1.8	5.0	64.30%	

ORR, objective response rate; DCR, disease control rate; mPFS, median progression-free survival; mOS, median overall survival;1L, first-line; 2L, second-line; 3L, third-line; ≥3L, third-line or beyond; FOLFIRI, 5-fluorouracil + irinotecan + calcium folinate.

To rigorously evaluate ramucirumab’s efficacy, investigators designed the pivotal phase III REGARD study. It evaluated the efficacy of ramucirumab versus placebo in treating advanced GC patients who had failed first-line platinum or fluoropyrimidine-based chemotherapy. A total of 355 patients were enrolled, with 238 assigned to the ramucirumab group and 117 to the placebo group. The mOS in the ramucirumab group was 5.2 months compared to 3.8 months in the placebo group, indicating statistically significant survival benefits. Regarding adverse events, the incidence of hypertension was slightly higher in the ramucirumab group (16% vs. 8%). The overall incidence of adverse events (94% vs. 88%) and the incidence of grade 3 or higher adverse events (57% vs. 58%) were similar between the two groups. These findings confirmed the manageable safety profile of ramucirumab (10).

Another landmark study, RAINBOW, further solidified the clinical significance of ramucirumab. This phase III trial covered 665 patients randomly assigned to receive either the combination of ramucirumab and paclitaxel (330 patients) or paclitaxel monotherapy (335 patients). The combination regimen notably prolonged OS in GC patients with failed first-line chemotherapy (9.6 months vs. 7.4 months). Although the overall incidence of adverse events was comparable between the two groups, the combination therapy group experienced a higher proportion (with a difference exceeding 10%) of severe adverse events. These predominantly included neutropenia (41% vs. 19%), leukopenia (17% vs. 7%), and hypertension (14% vs. 2%) (11).

To further delve into the potential of ramucirumab in the first-line treatment of GC, researchers carried out the phase III RAINFALL trial. This trial involved 126 centers across 20 countries and enrolled a total of 645 patients (326 patients in the ramucirumab plus fluorouracil and cisplatin group and 319 patients in the placebo plus fluorouracil and cisplatin group). The primary endpoint was defined as median progression-free survival (mPFS), while the secondary endpoint was mOS. The mPFS in the ramucirumab arm showed a slight increase compared to the placebo arm (5.7 months vs. 5.4 months, p=0.0106). However, the improvement in mOS did not reach statistical significance (11.2 months vs. 10.7 months, p=0.6757). Additionally, patients in the ramucirumab arm experienced a higher incidence of grade 3 hypertension (10% vs. 2%) and gastrointestinal perforation (4% vs. 1%). Based on these findings, the researchers concluded that routine addition of ramucirumab to platinum- and fluoropyrimidine-based chemotherapy was not advisable as first-line treatment for advanced GC (22).

At the same time, investigators conducted the prospective, randomized, double-blind, phase II RAINSTORM trial in East Asia. This trial enrolled 189 patients with previously untreated advanced GC, who were randomly assigned to receive ramucirumab combined with S-1 and oxaliplatin (SOX) (96 patients) or placebo combined with SOX (93 patients). Analysis revealed that adding ramucirumab to the SOX regimen did not significantly improve either PFS or OS (23). In 2022, Japanese investigators presented new evidence for the treatment of elderly GC patients through a prospective phase II study that assessed the efficacy of first-line S-1 combined with ramucirumab in an elderly cohort with advanced/recurrent GC. The study involved 48 patients with a median age of 77.5 years. The findings revealed a one-year OS rate of 63.7%, meeting the pre-specified primary endpoint. The mPFS and mOS were 5.8 months and 16.4 months. The most common grade 3–4 adverse events were neutropenia (27.7%), anorexia (23.4%), anemia (19.1%), hypertension (14.9%), leucopenia (12.8%), and hypoalbuminemia (12.8%). The combination of S-1 and ramucirumab was found to exhibit good anti-tumor efficacy and tolerable toxicity, leading the investigators to conclude that it could offer a novel treatment option for elderly patients with advanced or recurrent GC (24).

Currently, the standard second-line treatment for advanced GC is ramucirumab, either administered as monotherapy or in combination with paclitaxel. To further broaden its clinical application, the prospective phase III RAMIRIS trial is assessing the therapeutic efficacy of the FOLFIRI (5-fluorouracil + irinotecan + calcium folinate) regimen in combination with ramucirumab in patients with advanced GC who have experienced disease progression following first-line paclitaxel-based chemotherapy (28). Additionally, two clinical trials (NCT06169410, jRCTs05119071) are exploring the value of ramucirumab in the perioperative treatment of GC. These studies are expected to provide more evidence-based support for the use of ramucirumab in the management of GC.

#### 2.1.2 Bevacizumab

Bevacizumab, a recombinant humanized anti-angiogenic monoclonal antibody targeting VEGF, inhibits angiogenesis by binding to VEGF and blocking its interaction with VEGFR-1 and VEGFR-2. This drug has been approved for the treatment of multiple solid tumors, including metastatic colorectal cancer, lung cancer, breast cancer, ovarian cancer, endometrial cancer, and clear cell renal cell cancer. Regarding GC, researchers have carried out a series of phase II and phase III clinical trials to assess the efficacy of bevacizumab in combination with chemotherapy for patients with advanced GC. Outcomes of bevacizumab clinical trials are summarized in **Table 2**, demonstrating limited clinical benefits across various treatment settings.

**Table 2:** Summary of bevacizumab clinical trials in gastric cancer across multiple lines of therapy.

Treatment line	Phase	Registration number	Drugs (number)	ORR	DCR	mPFS (months) or PFS rate	mOS (months) or OS rate	Grade 3/4 adverse events	RO rate	References
1L	III	NCT00887822/AVATAR	Bevacizumab +Cisplatin+Capecitabine(100)	/	/	6.3	45% (1year)	8.00%	/	(29)
			Cisplatin+Capecitabine(102)	/	/	6	48% (1year)	15.00%		
	III	NCT00548548/AVAGAST	Bevacizumab+Chemotherapy(387)	46.00%	75.90%	6.7	12.1	/	/	(30)
			Placebo+Chemotherapy(387)	37.40%	67.70%	5.3	10.1	/		
	II	NCT00952003/AGMT_GASTRIC-3	Bevacizumab+Oxaliplatin+Irinotecan→Docetaxel+Bevacizumab(40)	51.52%	78.79%	7	11	/	/	(31)
	II	NCT01191697	Bevacizumab+Trastuzumab+XELOX(36)	81%	94.40%	14	23.2	/	/	(32)
	II	NCT01359397	Bevacizumab+XELOX+Docetaxel(60)	70%	96%	8.3	12	78%	/	(33)
	I/II	NCT00845884	Bevacizumab+Cisplatin+Capecitabine+Docetaxel(17)	41%	88%	7.6	13.9	82.00%	/	(34)
	II	NCT00673673	Bevacizumab+mFOLFOX6(39)	56.40%	92.30%	7.8	14.7	/	/	(35)
	II	/	Bevacizumab+Cisplatin+Irinotecan(34)	65%	/	/	12.3	/	/	(36)
	II	/	Bevacizumab+Trastuzumab+XELOX+Docetaxel(25)	74.00%	95.00%	10.8 47% (1year)	17.9 79%(1year)	/	/	(37)
≥3L	III	EudraCT:2018-004845-18.	TAS102+Bevacizumab(50)	8%	53%	3.9	9.3	74.00%	/	(38)
			TAS102(53)	2%	66%	3.1	8.5	66.00%		
	Review	/	Apatinib+Bevacizumab(45)	51.11%	91.11%	9.94	26.44	>17.78%	/	(39)
			Apatinib(55)	47.27%	96.36%	9.23	19.64	>20%		
Perioperative	II	/	Bevacizumab+Docetaxel+Capecitabine+Cisplatin(20)	55.00%	95.00%	/	100% (1year), 75.8% (2year), 75.8% (3year)	/	90%	(40)
	Review	/	Bevacizumab+Docetaxel+Oxaliplatin+5-FU(40)	65%	95.00%	/	16.4	/	75%	(41)
			Docetaxel+Oxaliplatin+5-FU(40)	42.50%	85.00%	/	17.6	/	50%	

In 2011, the pivotal phase III prospective AVAGAST trial enrolled 774 patients, who were randomly assigned in a 1:1 ratio to receive either bevacizumab combined with cisplatin and capecitabine or placebo combined with chemotherapy. The study aimed to assess the efficacy of bevacizumab in the first-line treatment of advanced GC. The trial results showed that adding bevacizumab to the chemotherapy regimen resulted in a slight improvement in OS (12.1 months vs.10.1 months, p=0.1002). Nevertheless, this improvement did not reach statistical significance (30).

Since only 12 Chinese patients were included in the AVAGAST study, in 2014, the investigators carried out the Phase III AVATAR study specifically targeting the Chinese population. A total of 202 Chinese patients were enrolled in this study, which adopted a design similar to that of AVAGAST. This study demonstrated that adding bevacizumab to the capecitabine-cisplatin regimen did not bring about a significant improvement in the prognosis of Chinese patients with advanced GC. The study showed that when bevacizumab was added to the capecitabine-cisplatin regimen, there was no statistical significance in either PFS (6.3 months vs. 6.0 months, p=0.47) or OS (10.5 months vs. 11.4 months, p=0.56) between the two groups (29).These results which did not meet expectations in the Chinese population further underscore the limited clinical utility of bevacizumab in GC.

In 2024, a prospective phase III clinical trial was conducted to assess the efficacy of the combination of Trifluridine-Tipiracil Hydrochloride Mixture (TAS102) and bevacizumab against TAS102 monotherapy. A total of 103 patients were enrolled and randomly assigned to receive either TAS102 (n=53) or the combination of TAS102 and bevacizumab (n=50). The findings revealed that, in comparison with TAS102 monotherapy, the addition of bevacizumab did not significantly prolong either PFS (3.9 months vs. 3.1 months, p=0.058) or OS (9.3 months vs. 8.5 months, HR 0.91) (38). This recent negative result reinforces the pattern of bevacizumab’s limited efficacy in post-line treatment and combination strategies.

Based on existing research evidence, adding bevacizumab to treatment regimens for GC patients did not achieve the expected clinical benefits. Additionally, bevacizumab showed limited therapeutic benefits in perioperative treatment of GC, as documented in **Table 2**. In recent years, research on bevacizumab in GC has relatively decreased, and therefore it has not been incorporated into the standard treatment regimen for patients with advanced GC.

ORR, objective response rate; DCR, disease control rate; mPFS, median progression-free survival; mOS, median overall survival;1L, first-line; 2L, second-line; 3L, third-line; ≥3L, third-line or beyond; XELOX, capecitabine + oxaliplatin.

#### 2.1.3 Aflibercept

Aflibercept, a fusion protein, binds VEGF-A, VEGF-B, and PLGF with high affinity. As a ligand trap, it blocks these growth factors, reducing neovascularization and vascular permeability to inhibit tumor growth (42, 43). Besides its approved applications in various ophthalmic disorders, such as wet age-related macular degeneration, macular edema secondary to retinal vein occlusion, and diabetic macular edema, aflibercept has also exhibited promising therapeutic potential in the treatment of colorectal cancer (44–46).

In the realm of GC treatment, a phase II trial in 2019 investigated the therapeutic efficacy of aflibercept combined with oxaliplatin, calcium folinate, and 5-fluorouracil (mFOLFOX6) as a first-line treatment for metastatic esophagogastric adenocarcinoma. However, the study did not meet its primary endpoint. The data revealed that aflibercept in conjunction with mFOLFOX6 did not significantly improve mPFS compared to placebo (9.7 months vs. 7.4 months, p=0.72), indicating that adding aflibercept to the mFOLFOX6 regimen did not augment its effectiveness (47). In 2020, the MOMENTUM trial a evaluated aflibercept plus capecitabine in advanced gastrointestinal and breast cancers, including eight patients with advanced GC. Although the sample size is small, the regimen initially showed a tolerable safety profile and demonstrated antitumor activity, offering valuable insights for future research (48).

#### 2.1.4 Pulocimab

Pulocimab, a fully human monoclonal antibody with specific binding affinity for VEGFR2, has demonstrated acceptable safety and promising antitumor activity in phase I clinical trials (49). An Ib/II phase clinical investigation presented at the 2024 annual meeting of the American Society of Clinical Oncology (ASCO) systematically evaluated the safety and efficacy of the combination regimen consisting of cadonilimab, pulocimab, and paclitaxel as a second-line treatment strategy for advanced GC patients. A total of 59 patients were randomly allocated to two treatment arms: the experimental group receiving cadonilimab in combination with pulocimab and paclitaxel (Group 1, n=29) and the control group receiving placebo plus pulocimab and paclitaxel (Group 2, n=20). Results showed that Group 1 has a higher objective response rate (ORR) (48.0% vs. 39.3%) and an extended mPFS (6.8 months vs. 4.9 months) than Group 2. In terms of safety, the most common grade 3–4 adverse events were neutropenia (27.6% vs. 33.3%), leukopenia (10.3% vs. 26.7%), and hypertension (13.8% vs. 0.0%) with no new safety concerns emerging during the study period (50). Collectively, these findings suggest that combining dual immunotherapies with VEGFR-2-targeted agents may overcome immunotherapy resistance, but large-scale, multi-center trials are needed to confirm long-term efficacy and safety.

### 2.2 Tyrosine kinase inhibitor

While monoclonal antibodies demonstrated efficacy in second-line therapy, their intravenous administration prompted the development of orally bioavailable small-molecule TKIs. These agents offered advantages in convenience and multi-target inhibition.

#### 2.2.1 Apatinib

Apatinib, a novel small-molecule VEGFR-2 inhibitor independently developed in China, acts by specifically binding to VEGFR-2 sites to block the phosphorylation of VEGFR-2, thereby inhibiting angiogenesis (51). A summary of previous clinical trials related to apatinib is presented in **Table 3**, showing efficacy and safety data across different treatment lines.

**Table 3:** Summary of apatinib clinical trials in gastric cancer across multiple lines of therapy.

Treatment line	Registration Number	Phase	Drugs (number)	ORR	DCR	mPFS (mounths) or PFS rate	mOS (mounths) or OS rate	RO rate	Grade 3/4 adverse events	References
≥3L	NCT02426034/AHEAD	IV	Apatinib(1999)	4.40%	35.80%	2.7	5.8	/	51.00%	(52)
	NCT03042611/ANGEL	III	Apatinib(308)	6.50%	40.30%	2.83	5.78	/	47.60%	(53)
			Placebo(152)	1.30%	13.20%	1.77	5.13	/	43.70%	
	/	III	Apatinib850mg(176)	2.84%	42.05%	2.6	6.5	/	/	(54)
	NCT00970138	II	Apatinib850mg(47)	0.00%	100.00%	3.2	4.27	/	/	(55)
			Apatinib425mg(46)	0.00%	50.00%	3.67	4.8	/	/	
			Placebo(48)	0.00%	17%	1.4	2.5	/	/	
	NCT03104283	II	Apatinib500mg(27)	16.70%	72.90%	2.8	5.4	/	50.00%	(56)
			Apatinib250mg(21)			3.47	8.13	/	13.90%	
	/	Review	Apatinib+S-1(42)	9.50%	71.40%	4.1	8.6	/	/	(57)
			S-1(42)	0.00%	40.50%	2.2	7.8	/	/	
	/	Review	Apatinib850mg(20)	10%	70%	3.5	4.5	/	19.00%	(58)
	NCT02668380/AHEAD-G202	Observational	Apatinib250mg(83) vs Apatinib425-500mg(135) vs Apatinib675-850mg(13)	14.00%	76.60%	4.2	7.13	/	/	(59)
	NCT03333967	Observational	Apatinib500/250mg(747)	6.83%	56.89%	5.56	7.5	/	18.97%	(60)
2L	NCT04345783	II	Apatinib+Camrelizumab+S-1(24)	29.20%	95.80%	6.5	75%(6months)/41.6%(1 year)	/	25.00%	(61)
	NCT04182724	II	Placebo(91)	0.00%	8.79%	1.8	4.7	/	/	(62)
			Anti-PD-1+Paclitaxel+Apatinib(43)	20.90%	88.30%	6.2	10.1	/	/	
	NCT04190745	II	Apatinib+Toripalimab(25)	20.00%	68.00%	2.77	8.3	/	24.00%	(63)
			Irinotecan/Paclitaxel/Docetaxel(26)	26.90%	80.80%	2.33	9.88	/	34.60%	
	NCT04338438	II	Apatinib+S-1(37)	21.60%	83.80%	4.2	8.2	/	21.60%	(64)
	/	review	Apatinib+S-1(63)	50.80%	74.60%	5.3	10.7	/	/	(65)
			S-1(63)	30.20%	54.00%	4.2	8.1	/	/	
1L	NCT02525237	II	Apatinib+S-1(30)	21.70%	78.30%	4.21	7.49	/	/	(66)
	NCT03472365	II	Apatinib+Camrelizumab+XELOX(48)	58.30%	93.75%	6.8	14.9	/	/	(67)
	NCT03244774	I	Apatinib+POF(21)	81.30%	87.50%	10.4	18.4	/	71.40%	(68)
	ChiCTR2000034109/SPACE	I	Apatinib+Camrelizumab(34)	76.50%	91.20%	8.4	69.1%(1year), 62.8%(2year)	/	52.90%	(69)
	/	/	Apatinib+S-1(35)	29.00%	93.50%	8.1	/	/	/	(70)
			S-1(34)	9.70%	67.70%	5	/	/	/	
Perioperative	NCT04208347/DRAGON IV/CAP 05	III	Camrelizumab +Apatinib+SOX(160)	/	/	/	/	64.70%	34%	(71)
			SOX(160)	/	/	/	/	63.90%	17%	
	NCT02529878/Ahead-G325	II	Apatinib+S-1/Paclitaxel(30)	73.30%	93.30%	/	83.4% (1year)	94.40%	58.10%	(72)
	NCT03878472	II	Camrelizumab +Apatinib+SOX(19)	28%	/	/	/	82.60%	0%	(73)
	NCT04195828	II	amrelizumab+Apatinib+Paclitaxel+S-1(51)	66.00%	/	/	/	94.10%	33.30%	(74)
			Paclitaxel+S-1(53)	43.40%	/	/	/	81.10%	26.40%	
	NCT03229096	II	Apatinib+Oxaliplatin+Capecitabine(32)	78.10%	/	42.6	69.4%(3year)	96.90%	56.25%	(75)
	ChiCTR2200055269	II	Apatinib+Sintilimab+FLOT(30)	85.70%	100.00%	/	96.4%(1year), 84.4%(2year)	/	33.30%	(76)
	ChiCTR-ONC-17010430	II	Apatinib+S-1+Oxaliplatin(37)	73.00%	81.10%	/	surgical group:71.1%(1year), 41.1%(2year)	63.60%	/	(77)
	NCT03192735	II	Apatinib+S-1+Oxaliplatin(48)	/	/	/	/	75%	33.30%	(78)
	/	/	Anti-PD-1+Apatinib+Chemotherapy(39)	74.40%	100.00%	3year: 76.1%	3year: 86.7%	/	/	(79)
			Apatinib+Chemotherapy(34)	58.80%	94.10%	3year: 46.9%	3year: 70.3%	/	/	

ORR, objective response rate; DCR, disease control rate; mPFS, median progression-free survival; mOS, median overall survival;1L, first-line; 2L, second-line; 3L, third-line; ≥3L, third-line or beyond; SOX, S-1 + oxaliplatin; RO rate, R0 resection rate; POF, paclitaxel + oxaliplatin + 5-fluorouracil; FLOT, 5- fluorouracil + leucovorin + oxaliplatin + docetaxel.

Apatinib was initially developed for the third-line treatment of advanced GC. In 2013, a prospective, randomized phase II trial enrolled 144 Chinese patients who were randomly assigned to receive placebo, 425 mg of apatinib, or 850 mg of apatinib. Compared with placebo, both apatinib doses significantly improved PFS and OS compared with placebo. The 425 mg group achieved PFS of 3.67 months and OS of 4.83 months, while the 850 mg group achieved PFS of 3.2 months and OS of 4.27 months (placebo: PFS 1.4 months, OS 2.5 months). Regarding safety, the drug was well tolerated by patients with serious adverse reactions mainly including hand-foot syndrome (2.1% vs. 4.3% vs. 13.4%) and hypertension (0% vs. 8.5% vs. 10.9%), and hematological toxicity was mostly moderate with minimal grade 3–4 hematological toxicity (55). These promising findings provided the foundation for subsequent phase III investigations in GC patients.

To substantiate these preliminary findings, the clinical significance of apatinib was further confirmed in a crucial Phase III study carried out in 2016. A total of 267 patients suffering from advanced GC were enrolled and randomly assigned to receive either apatinib (176 patients) or placebo (91 patients). Among patients with advanced GC who were resistant to second-line or multiple lines of chemotherapy, the apatinib group experienced a notable extension in both PFS (2.6 months vs. 1.8 months) and OS (6.5 months vs. 4.7 months). A subsequent examination of adverse events indicated that the incidence rates of grade 3–4 hand-foot syndrome (8.5% vs. 0%), hypertension (4.5% vs. 0%), and proteinuria (2.3% vs. 0%) were higher in the apatinib group than in the placebo group. Nevertheless, the overall safety situation remained within an acceptable and controllable range (54). These research results contributed to the approval of apatinib by the Chinese Drug Administration in 2014 for use in the third-line treatment of advanced GC.

The phase III ANGEL trial published in 2024 evaluated apatinib in third-line or ≥fourth-line advanced GC treatment. Among 460 patients (308 in apatinib group, 152 in placebo group), mOS improvement was not statistically significant (5.78 vs. 5.13 months, p=0.4724), but significant improvements were observed in mPFS, ORR, and disease control rate (DCR). Moreover, in patients receiving ≥ 4-line therapy, there were significant benefits in mOS (6.34 vs. 4.73 months) and mPFS (3.52 vs. 1.71 months). Regarding safety, the most common grade ≥ 3 adverse events were hypertension, anemia, elevated transaminases, malaise, and proteinuria, but the overall safety profile was manageable. The investigators attributed the lack of statistical significance in the primary OS endpoint to potential confounding by post-study anticancer therapies. Nevertheless, the significant improvements in median PFS, ORR, and DCR provide compelling evidence that this regimen warrants further validation through larger-scale randomized controlled trials (53).

In recent years, the combination of apatinib and PD-1 inhibitors has emerged as a key area of research. *In vitro* studies have established the synergistic anti-tumor effects of these two agents (80).A phase I clinical trial in 2019 assessed the safety and efficacy of the combination of SHR-1210 (an anti-PD-1 antibody) and apatinib in treating patients with advanced hepatocellular carcinoma, GC, or esophagogastric junction cancer. The findings showed that the mPFS of this combination reached 2.9 months and the mOS reached 11.4 months, which were superior to the outcomes of apatinib monotherapy, and preliminarily confirmed the synergistic effect of anti-PD-1 antibody and VEGFR2 inhibitor (81). Recent investigations have elucidated the underlying synergistic mechanism: apatinib can downregulate the expression of both the chemokine CXCL5 and the antigen CD74 within the tumor microenvironment. This leads to a decrease in the recruitment of tumor-associated neutrophils and an improvement in the immune microenvironment for anti-PD-1 immunotherapy, thereby enhancing the immunotherapeutic effect (82).

In 2021, several trials explored the value of combining apatinib with chemotherapy in the treatment of advanced GC. Trial data indicated that when used as first-line treatment, the combination of apatinib and S-1 was not superior to standard chemotherapy regimens. However, as second-line treatment, it had enhanced efficacy compared to S-1 alone, with manageable side effects (64–66). A subsequent study of immunological combination regimens found that the combination of apatinib, camrelizumab, and XELOX (capecitabine + oxaliplatin) had favorable efficacy (mPFS: 6.8 months, mOS:14.9 months) and manageable toxicity for the first-line treatment of GC (67). Phase II trials from 2022 to 2024 further confirmed the promising anti-tumor activity of combining apatinib with chemotherapy and PD-1 inhibitors in the second-line treatment of advanced GC (63, 83). A 2024 prospective phase I study showed that the first-line treatment of advanced GC with camrelizumab combined with apatinib and chemotherapy elicited favorable responses in both patients with high and low CPS scores, the ORR was 76.5% and the mPFS was 8.4 months (69).These results suggest that the combination of apatinib, immunotherapy, and chemotherapy has great potential in the treatment of advanced GC.

In the field of neoadjuvant therapy, the phase II Ahead-G325 trial in 2018 involved 30 patients with advanced GC to assess the efficacy and safety of combining S-1/paclitaxel with apatinib for neoadjuvant treatment of GC. The study results showed that this combination therapy had a favorable clinical effect, the ORR was 73.3%, the DCR was 93.3%, the resection rate was 94.4%, and the 12 months OS rate was 83.4% (72). In 2022, another phase II study evaluated the efficacy and safety of camrelizumab combined with apatinib and SOX in the neoadjuvant treatment of GC. The results showed a pathological complete response (pCR) was 15.8%, suggesting a significant correlation with biomarkers such as microsatellite instability status, PD-L1 expression level, and tumor mutational load (73). These promising pathological response rates have stimulated further research into neoadjuvant applications.

A prospective study in 2023 compared two treatment approaches for locally advanced GC. When compared with apatinib combination chemotherapy alone, the addition of a PD-1 inhibitor led to significant improvements in multiple outcomes. The PD-1 inhibitor group showed higher pathological remission rate (23.1% vs. 15.6%), 3-year PFS rate (76.1% vs. 46.9%), and 3-year OS rate (86.7% vs. 70.3%). The safety profile remained manageable (79). The 2024 phase III DRAGON IV/CAP 05 study further illustrated that the combination of camrelizumab, apatinib, and SOX for the perioperative treatment of GC significantly elevated the pCR rate (18.3% vs. 5%) with an acceptable safety margin (71). Collectively, these results suggest that the combination of apatinib, immunosuppressants, and chemotherapy could emerge as a new therapeutic option for GC patients during the perioperative period.

Moreover, at the 2024 ASCO-GI meeting, Zhang et al. reported the outcomes of a phase II trial exploring the combination of apatinib, camrelizumab, and SOX as first-line treatment for alpha-fetoprotein-producing gastric cancer (AFPGC). This trial involved a total of 36 patients and the ORR was 55.6%, DCR was 86.1%, the 12-months PFS rate was 42.1%, the 12-months OS rate was 63.7%. In terms of safety, the incidence of grade ≥3 adverse events was 36.1%, and no novel safety concerns emerged (84). Based on these results, the 2024 version of the Chinese Society of Clinical Oncology (CSCO) Guidelines for the Treatment of GC now clearly designates apatinib + camrelizumab + SOX as the preferred first-line therapy for AFPGC, representing a notable milestone in clinical management.

Numerous ongoing prospective trials are actively assessing the value of apatinib across various stages of GC, as summarized in **Table 4**, spanning from perioperative to advanced therapy. These studies are anticipated to yield more efficacious therapeutic strategies for GC patients, potentially revolutionizing the current treatment paradigm.

**Table 4:** Ongoing apatinib clinical studies in gastric cancer by treatment line.

Treatment line	Registration number	Phase	Patient population	Drug	Number of patients	Primary outcome measures
Perioperative	NCT03599778	I/II	Patients have received radical gastrectomy (D2, R0) for GC	XELOX+Apatinib vs XELOX	52	DFS
Perioperative	NCT03355612	/	Patients have received radical gastrectomy (D2, R0) for GC	XELOX+Apatinib vs XELOX	456	DFS
2L	NCT03889626	III	Patients with GC who received 5-FU based regimen fist line treatment and non-PD	Apatinib vs Capecitabine	242	PFS
2L	NCT03144843	II	Patients with GC who failed first-line therapy	Apatinib+Paclitaxel vs Placebo+Paclitaxel	110	PFS
2L	NCT02596256	II	Patients with GC who failed first-line therapy	Apatinib+Docetaxel vs Docetaxel	80	PFS
≥3	NCT05095636	II	Patients with GC who failed second-line therapy	Apatinib+Camrelizuma vs Apatinib	102	PFS
/	NCT04948125	II	GC patients who progressed after receiving anti-PD-1/PD-L1/CTLA-4 antibody therapy	Camrelizuma+Apatinib	20	ORR

ORR, objective response rate; PFS, progression-free survival; DFS, disease-free survival; 1L, first-line; 2L, second-line; 3L, third-line; ≥3L, third-line or beyond; XELOX, capecitabine + oxaliplatin.

#### 2.2.2 Fruquintinib

Following the demonstrated efficacy of apatinib in GC, fruquintinib emerged as another highly selective VEGFR inhibitor. Fruquintinib is a highly selective orally administrable small-molecule antagonist of VEGFR1, VEGFR2, VEGFR3 (85). In 2023, a prospective phase Ib/II clinical trial was carried out to assess the efficacy of fruquintinib in combination with paclitaxel as second-line therapy for advanced GC. The findings indicated that this combination regimen demonstrated an acceptable safety profile and promising therapeutic efficacy in patients who had progressed after standard first-line treatment, with a mPFS of 4.0 months and a mOS of 8.5 months. A subsequent safety assessment revealed that the most common grade ≥3 adverse events were neutropenia (64.3%) and leukopenia (28.6%) (86).

The FRUTIGA study was a randomized, double-blind, placebo-controlled phase III trial conducted across 35 centers in China. This study enrolled 703 patients with advanced GC, who had progressed following fluorouracil and platinum-based chemotherapy. Patients were randomly assigned to fruquintinib plus paclitaxel (n=351) or placebo plus paclitaxel (n=352). The trial results demonstrated that the fruquintinib combination significantly improved PFS (5.6 vs. 2.7 months) Additionally, it achieved higher ORR (42.5% vs. 22.4%) and DCR (77.2% vs. 56.3%). However, the improvement in OS did not reach statistical significance (9.6 months vs. 8.4 months, p=0.6064). Investigators attributed this to differences in subsequent treatments. Safety profiles were generally comparable between groups, but the fruquintinib group exhibited a slightly higher rate of grade ≥3 adverse events (86.9% vs. 63.3%). Overall, FRUTIGA established fruquintinib plus paclitaxel as an effective second-line option for advanced GC, providing clear PFS benefits and improved response rates with manageable toxicity (87).

Currently, fruquintinib has received marketing approval in China and Europe for the treatment of colorectal cancer, thereby laying a solid foundation for its application in the management of digestive tract tumors. In the realm of GC, the 2024 edition of the CSCO Guidelines for the Treatment of GC has integrated data from the FRUTIGA study, which underscores the clinical significance of this therapeutic regimen. However, given that the FRUTIGA study did not meet the primary endpoint of demonstrating a significant overall survival benefit, fruquintinib has not yet been approved for second-line treatment of GC. Despite this, the promising outcomes of the FRUTIGA study have spurred the initiation of several ongoing clinical trials (as presented in **Table 5**). These trials are investigating the use of fruquintinib as a monotherapy, as well as in combination with standard chemotherapy and PD-1 inhibitors.

**Table 5:** Ongoing fruquintinib clinical studies in gastric cancer by treatment line.

Treatment line	Registration number	Phase	Patient population	Drug	Number of patients	Primary outcome measures
1L	NCT06094868	II	Patients with untreated unresectable GC	Fruquintinib+XELOX+Sintilimab	45	PFS
1L	NCT05795296	/	Patients with untreated unresectable GC	Fruquintinib+Sintilimab	30	ORR
1L	NCT06158919	II	Patients with untreated unresectable GC	Fruquintinib+nivolumab/Sintilimab+XELOX	58	PFS
1L	NCT05177068	II	Patients with untreated unresectable GC	Fruquintinib+Sintilimab+SOX	42	RO
1L	NCT05122091	II	Patients with resectable or potentially resectable locally advanced GC	Fruquintinib+SOX	53	PRR
1L	NCT05914610	/	Patients with untreated unresectable GC	Fruquintinib+ Envafolimab +SOX	100	RO
2L	NCT06102785	II	Patients with GC who failed first-line therapy	Fruquintinib+TAS102	30	PFS
2L	NCT05625737	/	Patients with GC who failed first-line therapy	Fruquintinib+Sintilimab	29	ORR
2L	NCT05643677	II	Patients with GC who failed first-line therapy	Fruquintinib+Irinotecan	47	PFS

ORR, objective response rate; PFS, progression-free survival; PRR, pathologic complete response rate; RO, R0 resection rate; 1L = first-line; 2L = second-line; XELOX, capecitabine + oxaliplatin; SOX, S-1 + oxaliplatin.

#### 2.2.3 Regorafenib

Regorafenib, an oral TKI, inhibits the activity of multiple protein kinases that play crucial roles in tumor angiogenesis, tumorigenesis, and the regulation of the tumor microenvironment (88). Currently, regorafenib is approved for the third-line treatment of metastatic colorectal cancer and the second-line treatment of hepatocellular carcinoma. In preclinical studies related to GC, regorafenib exhibited remarkable antitumor activity in a mouse xenograft model of GC. Specifically, it was observed to decrease the phosphorylation of mixed tumor lysates and the expression of VEGFR-2 proteins. Moreover, in nearly all tested models, regorafenib effectively inhibited tumor growth, angiogenesis, and the proliferation of tumor cells. These findings provide a robust scientific basis for the clinical application of regorafenib in GC treatment (89).

The INTEGRATE I study, carried out in 2016, was an international multicenter phase II prospective randomized clinical trial that enrolled 147 patients with advanced GC, with 97 patients receiving regorafenib treatment and 50 patients administered placebo. The study results showed that regorafenib significantly extended PFS in refractory advanced GC (2.9 months vs. 0.9 months), yet the improvement in mOS was not statistically significant (5.8 months vs. 4.5 months, HR, 0.74, P = 0.147). The adverse events observed were in line with previous reports, indicating a manageable safety profile (90). To confirm these promising signals in a larger cohort, the investigators conducted the phase III INTEGRATE IIa trial and reported the outcomes in 2024. This trial enrolled 251 patients with advanced GC from various regions and demonstrated that regorafenib significantly improved both PFS (HR, 0.53, p < 0.0001) and OS (4.5 months vs. 4.0 months, HR, 0.68, p=0.006) (91).

Regarding combination therapy approaches, researchers have explored the synergistic effects of regorafenib when combined with immunotherapy. The 2020 Phase Ib REGONIVO study was the first to assess the efficacy of regorafenib in combination with nivolumab as third-line therapy. In patients with GC, this combination achieved an ORR of 44%, thereby showcasing significant antitumor activity (92). A phase II trial in 2023 advanced this therapeutic strategy to first-line treatment, demonstrating that treating advanced GC patients with regorafenib in combination with nivolumab and chemotherapy led to an ORR of 76%, a mPFS of 13.0 months, and superior 12-month PFS and OS rates (51% and 85%) compared to previously reported regimens of chemotherapy combined with nivolumab (93–95). The findings of this study suggest that the combination of regorafenib and immunotherapy strategies holds great potential in the treatment of GC across all stages of disease progression.

The results of the previously discussed studies suggest that regorafenib has demonstrated both effectiveness and safety in the treatment of advanced GC. Currently, a phase III randomized controlled trial (NCT04879368) is being conducted to assess its application in the third-line treatment of advanced GC. Additionally, another phase II trial (NCT03627728) is investigating its potential for second-line treatment. The outcomes of these studies are expected to offer further evidence-based medical support for the clinical utilization of regorafenib in the management of GC.

#### 2.2.4 Anlotinib

Anlotinib is an oral TKI with multi-targeted properties, selectively targeting VEGFR, FGFR, and PDGFR. These targeted actions play a crucial role in inhibiting tumor angiogenesis and tumor cell proliferation (96–98). Initially, anlotinib demonstrated clinical applications in the treatment of Non-Small Cell Lung Cancer, medullary thyroid carcinoma, and soft tissue sarcoma (99–101). In recent years, its potential in the treatment of GC has increasingly been explored (102, 103). Preclinical studies have shown that anlotinib not only suppresses VEGF/PDG-BB/FGF-2 induced cell migration and capillary-like tube formation in endothelial cells but also reduces the expression of PD-L1, thereby providing a theoretical foundation for its combination with immunosuppressive agents (96, 104).

A Chinese prospective phase II study in 2022 investigated the value of anlotinib combined with toripalimab as second-line treatment for advanced GC. Enrolling 63 patients, the study showed an ORR of 32.3%, DCR of 91.9%, mPFS of 4 months, and mOS of 11.1 months. Notably, only 11.3% of patients experienced grade ≥3 adverse events, indicating that this combination regimen has manageable safety profiles (105). A Chinese retrospective study also revealed that anlotinib monotherapy or combination therapy demonstrated certain efficacy in the third-line treatment of advanced GC, with a mPFS of 3.0 months and a mOS of 6.0 months (106).

Another prospective phase II study called OASIS in 2024 further appraised the value of nivolumab combined with anlotinib for second-line and subsequent treatment of advanced gastric adenocarcinoma and esophageal squamous cell carcinoma. Involving 45 patients with advanced gastric adenocarcinoma and 3 patients with esophageal squamous cell carcinoma, the study revealed an ORR of 29.2%, DCR of 64.6%, mPFS of 4.0 months, mOS of 11.1 months, and 16.7% incidence of grade 3–4 adverse events. The nivolumab-anlotinib combination regimen showed clinical activity with manageable toxicity (107).

A comprehensive review of the existing literature indicates that anlotinib has shown therapeutic efficacy and a tolerable safety profile in the second-and third-line treatment of advanced GC. To further elucidate its clinical significance, several ongoing studies (NCT02461407, NCT05029102, NCT04713059) are evaluating the application of anlotinib across various treatment stages of GC. It is anticipated that these investigations will offer novel alternatives for the precision treatment of GC.

#### 2.2.5 Sunitinib

Sunitinib, a kind of TKI, has received approval for the treatment of gastrointestinal mesenchymal stromal tumors and renal cell carcinomas. Its mechanism of action involves suppressing the activity of multiple receptor tyrosine kinases, such as VEGFR, PDGFR, and FMS-like tyrosine kinase 3 (FLT3) (108).

In the early exploration of GC treatment, a prospective phase II study in 2011 assessed the efficacy and safety of sunitinib monotherapy as a second-line treatment approach. This study (n=78) demonstrated an ORR of 2.6%, DCR of 32.1%, mPFS of 2.3 months, and mOS of 6.8 months in patients with advanced GC (109). Simultaneously, another prospective phase II trial that involved 51 GC patients receiving second-line sunitinib monotherapy demonstrated a mPFS of 1.3 months and a mOS of 5.8 months (110). The outcomes of these two studies suggest that sunitinib monotherapy demonstrated modest clinical activity when used as a second-line treatment for advanced GC.

Considering the limited efficacy of single-agent therapy, researchers have delved into the potential of combination therapy strategies. Initially, a comprehensive assessment was conducted on the efficacy and safety of sunitinib combined with FOLFIRI as second-and third-line treatments. Although there was a positive trend in OS, the combination therapy did not reach the pre-specified primary endpoint (111).In the exploration of first-line therapy, a prospective phase I trial in in 2013 determined the Maximum Tolerable Dose (MTD) of sunitinib in combination with FOLFIRI to be 25 mg/day (112). Two subsequent phase I trials from Japan and Korea further verified this dosage, demonstrating that the combination regimen had a manageable safety profile and showed preliminary antitumor activity (113, 114). However, these preliminary results necessitate further validation through larger-scale clinical trials to elucidate the clinical value and optimal application mode of sunitinib in the treatment of GC.

#### 2.2.6 Pazopanib

Pazopanib, a kind of TKI that specifically targets VEGFR-1/2/3, has currently obtained approval for the treatment of advanced renal cell carcinoma and soft tissue sarcoma (115, 116). In the domain of GC treatment, a phase II trial in 2016 investigated the therapeutic effectiveness of pazopanib combined with the XELOX regimen for the first-line treatment of GC. The findings indicated that this regimen demonstrated certain efficacy, with a mPFS of 6.5 months and a mOS of 10.5 months. The safety profile of this regimen showed that 51.5% of patients experienced grade 3 or higher adverse reactions, including neutropenia, anemia, thrombocytopenia, and loss of appetite (117).

In 2022, another phase II clinical trial was carried out to assess the efficacy of pazopanib combined with chemotherapy (5-fluorouracil plus oxaliplatin) compared with chemotherapy alone as the first-line treatment for patients with advanced GC. The outcomes of this study showed that although the combination regimen presented certain signs of effectiveness, it did not show substantial clinical advantages (118). At present, research on the use of pazopanib in the treatment of GC is rather scarce, and its clinical application value still requires further exploration and verification through a greater number of studies.

#### 2.2.7 Sorafenib

Sorafenib is an orally administered multi-target TKI. Its molecular targets encompass the RAF kinase family (including RAF-1, the wild-type and V600E mutant B-RAF), VEGFR-1/2/3, along with PDGFR-β, c-KIT, and FLT3. Through the simultaneous inhibition of two crucial signaling pathways, namely the Raf/MEK/MAPK and VEGF pathways, sorafenib can efficiently suppress tumor cell proliferation, impede angiogenesis, and trigger apoptosis, thereby curbing tumor growth (119). Presently, sorafenib has been granted approval by the US FDA for the treatment of unresectable hepatocellular carcinoma, advanced renal cell carcinoma, and radioactive iodine-refractory differentiated thyroid cancer.

In 2020, a prospective phase II study called ECOG5203 furnished substantial data regarding the application of sorafenib in the treatment of GC. This study involved 44 patients with advanced GC who had not undergone previous chemotherapy, and it assessed the efficacy of the combination of sorafenib with docetaxel and cisplatin regimens. The results of the study showed that this combination regimen achieved an ORR of 41%, a DCR of 73%, a mPF of 5.8 months, and a mOS of 13.6 months. A subsequent safety assessment revealed that the adverse effect profile of this combination regimen was similar to that of previous docetaxel combined with cisplatin regimens, indicating that the safety profile was manageable (120).

In the same year, a prospective phase I dose-escalation trial conducted by Korean scholars determined the maximum tolerated dose of sorafenib when combined with capecitabine and cisplatin for the first-line treatment of advanced GC patients (121). Leveraging the findings of this initial study, the research team launched the prospective phase II STARGATE trial, with results reported in 2023. Although the combination regimen exhibited a manageable safety profile, it did not confer significant therapeutic benefits. Following a median follow-up of 12.6 months, the sorafenib combination chemotherapy group and the chemotherapy-alone group showed comparable ORR (54% vs. 52%), and no statistically significant differences were noted in mPFS (5.6 months vs. 5.3 months) or mOS (11.7 months vs. 10.8 months) (122).

Additionally, a phase I study was carried out to explore the potential advantages of combining sorafenib with S-1 and cisplatin as a first-line treatment modality for advanced GC. Comprising 13 patients, the study revealed that five patients achieved partial remission, while eight patients attained disease stabilization, without the emergence of novel specific or severe adverse reactions. Despite the small sample size, these findings suggest this regimen is tolerable and warrants evaluation in larger trials (123).

In the realm of third-line therapy, a phase II study was executed to assess the efficacy of sorafenib monotherapy in patients with advanced GC. Among the 34 patients enrolled in this study, the mPFS was 3.6 months, and the mOS was 9.7 months. Although the 8-week PFS rate (61%) of the trial did not meet the prespecified endpoint, considering the current situation where third-line treatment alternatives for advanced GC are scarce, these findings still hold considerable value (124).

In conclusion, the existing research findings regarding sorafenib in the treatment of GC have not demonstrated substantial benefits. This indicates that more extensive and in-depth investigations are warranted. Such studies should aim to explore the therapeutic value of sorafenib in patients with specific molecular subtypes and to refine combination therapy strategies for improved clinical outcomes.

#### 2.2.8 Lenvatinib

Lenvatinib, a multi-target TKI, exerts anti-angiogenic and anti-tumor effects by inhibiting multiple targets such as VEGFR-1/2/3, FGFR1-4, PDGFRα/β, RET, and c-KIT (125, 126). In preclinical research, lenvatinib has been demonstrated to significantly suppress the growth of GC patient-derived xenografts (PDX) and decrease intratumorally blood vessel density (127). Subsequent preclinical PDX studies further confirmed that the combination of lenvatinib with immune checkpoint inhibitors exhibited more potent antitumor activity compared to monotherapy, thus providing a theoretical basis for subsequent clinical investigations (128).

Regarding clinical studies, the prospective phase II EPOC1706 study assessed the efficacy and safety of lenvatinib combined with pembrolizumab for the first or second-line treatment of advanced GC. Enrolling 29 patients, the study showed an ORR of 69%, a mPFS of 7.0 months, but the mOS had not been reached. The most prevalent grade 3 treatment related adverse events were hypertension (38%), proteinuria (17%), and decreased platelet count (7%) (129). These results indicate the potential clinical utility of this combination regimen.

In 2024, a phase I clinical trial investigated the efficacy of zimberelimab (GLS-010, a PD-1 antibody) in combination with lenvatinib and XELOX for the first-line treatment of AFPGC. The study results showed a mPFS of 7.67 months and a mOS of 13.17 months, indicating that AFPGC patients may benefit from this therapeutic regimen. Regarding safety, the most frequently reported adverse reactions were fatigue (55.6%), hand-foot syndrome (55.6%), and rash (55.6%), with no grade 4 or higher adverse events observed (130). Building on these preliminary findings, a prospective phase II clinical trial (NCT06383559) is currently in progress to assess the efficacy and safety of lenvatinib in AFPGC patients, which is anticipated to further validate its clinical value.

### 2.3 Recombinant human vascular endothelial inhibitor (Endostar)

Endostar is a modified recombinant human vascular endothelial inhibitor with nine amino acid residues added to its N-terminus to improve protein stability and biological activity. As an anti-angiogenic pharmaceutical agent, it primarily functions by inhibiting the VEGF signaling pathway and the expression of Matrix metalloproteinases (MMPs) by binding to integrins (such as α5β1) on the surface of endothelial cells. This process enables it to inhibit tumor neovascularization and tumor cell migration and invasion (131–133). The clinical application of endostar was established in 2004 through a multicenter, randomized, double-blind, placebo-controlled phase III clinical study conducted in China. The study demonstrated that the combination of endostar with vincristine and cisplatin for the treatment of primary or recurrent advanced non-small cell lung cancer resulted in a significant improvement in ORR (35.4% vs. 19.5%), mPFS (6.3 months vs. 3.6 months), and mOS (14.87 months vs. 9.90 months) when compared with the chemotherapy + placebo group (134). Subsequent to these findings, endostar was approved by the China Food and Drug Administration in 2005 for the treatment of Non-Small Cell Lung Cancer (135).

Endostar has exhibited promising clinical potential in the treatment of GC. A randomized controlled trial was carried out to assess the efficacy of endostar combined with the SOX regimen as first-line therapy for advanced GC. A total of 165 patients were enrolled and randomly allocated to either the endostar + SOX group (n=80) or the SOX monotherapy group (n=85). The findings demonstrated that the combination regimen significantly prolonged PFS (15.0 months vs. 12.0 months) and enhanced the DCR (85% vs. 72.9%). The most prevalent grade 3–4 adverse events in both groups was myelosuppression(20% vs. 20%) (136). Analogously, in another investigation involving GC patients with liver metastases, the endostar + SOX group (n=30) showed significant improvements in ORR (63.3% vs. 43.3%), DCR (86.7% vs. 73.3%), and mPFS (15.3 months vs. 12 months) when compared with the SOX monotherapy group (n=30), while maintaining a comparable adverse effect profile (137).

A retrospective study in 2022 analyzed 90 patients with GC and malignant ascites. Endostar combined with intraperitoneal chemotherapy significantly enhanced several crucial metrics compared to chemotherapy alone. Specifically, mPFS increased from 8.1 months to 9.7 months, ORR rose from 54.7% to 75.7%, and DCR climbed from 75.5% to 94.6%. Significantly, there was no substantial difference in the incidence of adverse reactions between the two groups. The most common grade 3 and higher adverse events were myelosuppression (18.9% vs. 24.5%) and peripheral neurotoxicity (10.8% vs. 11.3%) (138). From this analysis, it can be concluded that although endostar has shown some clinical advantages in treating advanced GC, the number of relevant study reports remains relatively limited. As a result, it has not been incorporated into the standard treatment protocols for advanced GC patients yet. **Table 6** provides a comparative overview of key clinical trials across different anti-angiogenic agents. Due to heterogeneity in treatment lines and study designs, direct cross-trial comparisons should be interpreted with caution.

**Table 6:** Comparative summary of key clinical trials evaluating anti-angiogenic agents in gastric cancer.

Treatment line	Phase	Registration number	Drugs (number)	ORR	DCR	mPFS(months)	mOS(months) or OS rate	Grade3/4 adverse events	References
1L	III	NCT02314117/RAINFALL	Ramucirumab+Cisplatin+5-FU(326)	41%	82%	5.72	11.2	/	(22)
	II	NCT02539225/RAINSTORM	Oxaliplatin/S-1 +Ramucirumab(96)	58%	91%	6.34	14.65	29%	(23)
	III	NCT00887822/AVATAR	Bevacizumab +Cisplatin+Capecitabine(100)	/	/	6.3	45%	8%	(29)
	III	NCT00548548/AVAGAST	Bevacizumab+ Chemotherapy(387)	46%	76%	6.7	12.1	/	(30)
2L	III	NCT00917384/REGARD	Ramucirumab(238)	3%	49%	2.1	5.2	57%	(10)
	III	NCT01170663/RAINBOW	Ramucirumab+Paclitaxel(330)	28%	80%	4.4	9.6	/	(11)
	II	NCT03472365	Apatinib +Camrelizumab +XELOX(48)	58%	94%	6.8	14.9	/	(67)
	III	NCT03223376/FRUTIGA	Fruquintinib + Paclitaxel(351)	43%	77%	5.6	9.6	87%	(87)
≥3L	III	EudraCT:2018-004845-18.	TAS102+Bevacizumab(50)	8%	53%	3.9	9.3	74%	(38)
	IV	NCT02426034/AHEAD	Apatinib(1999)	4%	36%	2.7	5.8	51%	(52)
	III	NCT03042611/ANGEL	Apatinib(308)	7%	40%	2.83	5.78	48%	(53)
	III	NCT02773524/INTEGRATE IIa	regorafenib(169)	/	/	/	4.5	/	(91)

1L, first-line; 2L, second-line; 3L, third-line; ≥3L, third-line or beyond; XELOX: capecitabine + oxaliplatin.

## 3 Progress in the study of biomarkers for anti-angiogenic therapy in gastric cancer

The application of anti-angiogenic drugs has shown remarkable clinical efficacy in the treatment of GC. However, owing to tumor heterogeneity and individual differences, patient responses vary substantially. The precise identification of patient populations that will benefit from these drugs has emerged as a significant challenge. Discovering specific biomarkers is crucial for optimizing treatment strategies, enhancing efficacy prediction accuracy, and reducing the burden of adverse reactions in patients unlikely to benefit. Unfortunately, no well-established biomarkers currently exist to reliably guide the clinical use of anti-angiogenic agents. This review will explore the current status of biomarker research from three aspects: circulating biomarkers, tissue-based markers, and multi-omics models.

### 3.1 Circulation biomarkers

Compared with tissue biopsy, these biomarkers possess the advantages of being easily accessible, minimally invasive, and enabling dynamic monitoring. Consequently, they have garnered substantial attention in the research domain of anti-angiogenic therapy for GC. This provides an ideal platform for assessing treatment response and predicting which patients will benefit. Current investigations have been centered on the VEGF family and its associated molecules, the angiopoietin family, as well as other novel biomarkers.

The VEGF signaling pathway is a crucial target of anti-angiogenic therapy. Thus, members of the VEGF family and their receptors have great potential as biomarkers for predicting the efficacy of anti-angiogenic treatment. Research has indicated that elevated baseline serum levels of VEGF-A are associated with shortened OS. In contrast, increased levels of soluble VEGFR-2 are correlated with extended PFS and OS, implying a potentially positive response to anti-angiogenic therapy (139). However, the REGARD study, which assessed the therapeutic effect of ramucirumab, revealed that serum levels of VEGF-C, VEG-D, and soluble VEGFR1 and VEGFR3 did not exhibit statistically significant correlations with therapeutic benefit. This suggests that a single marker from the VEGF pathway may be insufficient for accurately predicting treatment outcomes (140). These correlative findings, while providing insights into potential response patterns, highlight that current biomarker-outcome relationships remain largely descriptive rather than mechanistically explanatory. The AVAGAST trial further investigated predictive markers in bevacizumab treatment and demonstrated that patients with lower baseline plasma levels of VEGF-A and neuropilin-1 (NRP-1) expression may experience greater improvements in OS (30).

Besides the VEGF family, several other angiogenesis-related molecules have shown predictive significance. Angiopoietin-2 (Ang-2), which acts as a ligand for the TIE2 receptor, is of great importance in the processes of vascular remodeling and the maintenance of vascular stability (141). Studies have shown that the baseline serum level of Ang-2 can serve as a predictor of OS in GC patients with advanced liver metastases. Additionally, it is closely related to lymph node metastasis in patients with early-stage GC (142, 143). Moreover, it has been observed that serum levels of soluble TIE2 are positively correlated with tumor progression. This implies that soluble TIE2 has the potential to be a biomarker for monitoring disease progression and assessing the therapeutic response in GC patients (144).

Although quite a few promising blood circulation markers have been identified, most of them are still in the preliminary research stage and have not been integrated into a systematic predictive model for clinical use. In the future, large-scale prospective studies are needed to further validate the predictive value of these biomarkers and explore biomarker combination strategies to improve prediction accuracy.

### 3.2 Tissue markers

In the treatment of GC, there are already well-recognized tissue markers like HER2, MSI, and PD-L1 that are used to guide targeted therapy and immunotherapy. Additionally, new markers such as Claudin18.2 and FGFR2b have emerged in the realm of targeted therapy research. However, the clinical utility of these markers in predicting anti-angiogenic therapy efficacy remains insufficiently validated.

VEGFR2, a crucial target in anti-angiogenic therapy, has received substantial attention because of the relationship between its tissue expression level and therapeutic efficacy. A retrospective analysis in the REGARD study showed that, regardless of the VEGFR2 protein expression level, the ramucirumab group tended to have improved OS and PFS compared to the placebo group. This suggests that the VEGFR2 expression level may not be the only decisive factor. Meanwhile, the study also found that the efficacy advantage of ramucirumab in HER2 positive patients was less significant than that in HER2 negative patients. However, due to the small sample size of HER2 positive samples (n=12), this conclusion needs to be verified by a larger-scale study (140). This finding is in line with another study in 2021, which confirmed that HER2 overexpression is closely related to neovascularization in GC and is an independent predictor of GC prognosis, providing a theoretical basis for exploring the relationship between HER2 expression status and the optimization of anti-angiogenic treatment strategies (145).

Moreover, recent studies have found several new targets, such as Thymosin β10 (TMSB10), Neuro-Oncological Ventral Antigen 2 (NOVA2), Granulocyte colony-stimulating factor (G-CSF) and G-CSFR. These targets have been shown to have the ability to act as predictive biomarkers (146–148).

In summary, the research on tissue markers for anti-angiogenic therapy is relatively lagging behind and faces multiple challenges such as tumor heterogeneity, difficulty in sampling, and the imperative for standardized testing procedures. Current tissue markers have demonstrated potential predictive significance in the anti-angiogenic treatment of GC. However, large-scale prospective clinical trials are still required to validate their effectiveness. Future investigations should focus on formulating standardized detection methodologies and evaluation criteria. Additionally, there should be an exploration of the value of combining these tissue markers with other predictive tools to enhance the accuracy of predicting treatment responses and patient outcomes in anti-angiogenic therapy for GC.

### 3.3 Multi-omics modeling

Considering that individual circulating or tissue markers have a restricted predictive value, researchers have started to explore more systematic prediction strategies based on multi-omics data. By integrating multidimensional data from genomics, transcriptomics, proteomics, metabolomics, and other relevant fields, researchers have obtained a more comprehensive understanding of the molecular typing and precise treatment of GC (149–152). The multi-omics approach has been proven to surmount the drawbacks of single markers and can more comprehensively reflect the biological characteristics and heterogeneity of tumors. This offers new insights for the precise implementation of anti-angiogenic therapy.

In the research domain of GC, substantial advancements have been achieved in the development of multi-omics approaches. Li et al. carried out a single-arm, phase II exploratory trial (NCT03878472) to assess the efficacy of camrelizumab combined with apatinib and chemotherapy regimens for the treatment of cT4a/bN+ stage GC. Simultaneously, multi-omics analysis uncovered several potential biomarkers, such as mutations in RREB1 and SSPO, immune-related characteristics, and peripheral T-cell expansion scores. This discovery emphasizes the crucial role of multi-omics in identifying candidate biomarkers (73). In a related investigation, Zeng et al. devised a multi-omics-based model named TMEscore. The objective of this model is to enhance the precision of treatment for advanced GC by utilizing the quantitative analysis of tumor microenvironmental characteristics (153). The model incorporates multiple microenvironmental factors to more accurately forecast the efficacy of immune-combination anti-angiogenic therapy, and it is anticipated to serve as an important reference tool for clinical decision-making.

In a phase II clinical trial aimed at evaluating the neoadjuvant therapy consisting of camrelizumab combined with chemotherapy, Zhao et al. conducted multi-omics analysis and discovered a significant association between HER2 positivity, CTNNB1 mutation, treatment sensitivity, and favorable prognosis which establishes a crucial foundation for the future development of precise neoadjuvant treatment regimens (154).Chen et al. utilized artificial intelligence techniques to develop the multimodal deep learning model (MuMo) that demonstrated remarkable performance in predicting the response to anti-HER2 monotherapy and combination immunotherapy among HER2-positive GC patients with area under the curve (AUC) values of 0.821 and 0.914 respectively and successfully identified a subgroup of low-risk patients with a good prognosis (155). Although the primary objective of this model is anti-HER2 therapy, its approach of integrating multi-omics data provides valuable insights and technical references for predicting the efficacy of anti-angiogenic therapies.

These studies have illustrated the substantial potential of multi-omics technology in improving the assessment, prediction, and personalized treatment of GC response. However, technical hurdles, including data integration and standardization, still impede its clinical translation. Hence, future prospective studies should further validate the application value of multi-omics prediction models in anti-angiogenic therapy and explore the potential of multi-omics in elucidating drug resistance mechanisms, thereby offering novel directions for the implementation of personalized treatment strategies.

## 4 Challenges

Although anti-angiogenic drugs have currently shown efficacy advantages in the treatment of advanced GC, several critical challenges still need to be overcome.

Firstly, the therapeutic efficacy of existing anti-angiogenic drugs is limited, and few medications have received regulatory approval. Despite the significant milestone marked by the approval of bevacizumab for advanced colorectal cancer in 2004 (156), investigations into first-line anti-angiogenic therapy for GC have not achieved the anticipated outcomes. Studies including AVATAR, AVAGAST, RAINFALL, and aflibercept trials have not achieved the anticipated outcomes (4, 22, 29, 47). Additionally, small molecule TKIs such as pazopanib and sorafenib have also been unsuccessful in demonstrating efficacy. Currently, only ramucirumab has proven effective in second-line therapy, as evidenced by trials like REGARD and RAINBOW (10, 11).Apatinib has been recommended for third-line therapy and first-line therapy in AFPGC. The FRUTIGA study demonstrated that fruquintinib in combination with paclitaxel, although improving second-line PFS in GC, was not approved due to failure to meet the OS benefit endpoint (87).

Secondly, the mechanisms underlying drug resistance are intricate and formidable to overcome. Tumor cells develop acquired or intrinsic resistance to anti-angiogenic drugs via diverse mechanisms. These mechanisms include adaptive upregulation of proangiogenic factors, stromal cell aggregation and recruitment, tumor microenvironment alterations, vascular co-option, vascular mimicry, and vascular intussusception (157, 158). Notably, some gastric tumors exhibit non-angiogenic phenotypes that bypass VEGF-dependent neovascularization entirely. Through vascular co-option, tumor cells exploit pre-existing vasculature, while vasculogenic mimicry enables tumor cells to form functional vascular channels independently of endothelial cells (159, 160).These non-angiogenic mechanisms represent intrinsic resistance pathways that allow tumors to maintain blood supply without relying on VEGF signaling, thereby limiting the efficacy of anti-angiogenic therapies in certain gastric cancer patients.

Moreover, tumor and vascular heterogeneity significantly contribute to the development of drug resistance. For instance, in the case of bevacizumab, acquired resistance restricts its efficacy to only 6–14 months. Although it can prolong PFS, the improvement in OS is minimal (see **Table 2**). This limited therapeutic benefit is a common trend observed across the majority of studies on anti-angiogenic therapy.

Furthermore, there is a lack of validated predictive biomarkers. The challenges in biomarker development are highlighted by the consistent failures, including REGARD, AVAGAST and other bevacizumab and ramucirumab studies. These collective failures emphasize the inadequacy of single-pathway biomarkers and the urgent need for more sophisticated predictive approaches in anti-angiogenic therapy. Although technological advancements have revealed the potential of circulating angiogenic factors, circulating tumor cells, and blood perfusion CT, none of these markers have been clinically validated for routine use (161–163). The absence of reliable efficacy predictive markers not only impedes the implementation of precision therapy but also obstructs a more profound understanding and the surmounting of drug resistance mechanisms. However, the development of biomarkers remains confronted with substantial challenges, primarily due to the complexity of tumor angiogenesis, tumor heterogeneity, the unpredictability of treatment efficacy and toxicity, and the constraints of clinical trials.

## 5 Potential strategies

In response to the above-mentioned challenges, the future development of anti-angiogenic therapy for GC may focus on the following directions:

Firstly, combination therapy strategies represent as a crucial approach to surmounting the current challenges in anti-angiogenic therapy for cancer. The combination of angiogenesis inhibitors and immune checkpoint inhibitors exhibits multiple synergistic mechanisms. These include reversing VEGF-mediated immunosuppression, promoting vascular normalization, and enhancing immune cell infiltration into the tumor microenvironment. Additionally, this combination increases sensitivity to anti-angiogenic agents and extends therapeutic response duration (157, 164). Based on these theoretical foundations, multiple clinical trials of immune combined anti-angiogenic therapy are underway. In addition, through combination chemotherapy, radiotherapy, cancer vaccines and other treatment methods, it is also expected to overcome drug resistance and enhance anti-tumor effects (165–167).

Secondly, novel bispecific antibodies offer an innovative approach in the realm of drug development. Ivonescimab, a bispecific antibody targeting both VEGF-A and PD-1, exerts its antitumor effects by competitively blocking the binding of VEGF-A and PD-1 to their respective ligands (168). Clinical investigations have evidenced the efficacy of this agent not only among patients with high PD-L1 expression but also those who derive limited benefit from conventional immunotherapy. These studies further demonstrate that such bispecific antibodies are effective in both high and low PD-L1 expression populations, without a substantial increase in adverse events (169). This class of bispecific antibodies has been shown to broaden the applicable patient population, concurrently enhancing both the immune microenvironment and the tumor microvascular environment, thereby underscoring its considerable research significance.

Thirdly, the development and optimization of drug delivery strategies represent crucial research areas. Current research endeavors primarily center on the creation of drugs with novel mechanisms of action, the refinement of administration timing and dosage regimens, and the exploration of synergistic interactions among different medications. Prospective clinical trials, such as DRAGON IV/CAP 05 and NCT03355612, are assessing the value of perioperative applications of these strategies, while studies like NCT03889626 are concentrating on innovative approaches for second-line treatment. It is imperative to place particular emphasis on evaluating dose tolerance and monitoring adverse reactions. The overarching goal is to ensure treatment efficacy while minimizing toxic side-effects. It is expected that the optimization of combination regimens involving anti-angiogenic drugs, chemotherapy, and other targeted agents will enable the overcoming of the current limitations in therapeutic efficacy.

Finally, biomarker research is indispensable for the realization of precision therapy in GC. Future investigations in this area will predominantly involve integrated multi-omics analyses, constructing more comprehensive prediction models by combining multi-dimensional data from genomics, transcriptomics, proteomics, and other relevant fields. Future research should consider the molecular typing and pathological characteristics of GC to offer personalized treatment strategies for different subtypes of patient populations. For example, Li et al. identified potential biomarkers such as RREB1 and SSPO mutations, immune-related features, and peripheral T-cell expansion scores through multi-omics analysis in a single-arm, phase II exploratory trial (NCT03878472), providing a solid foundation and predictive indicators for the anti-angiogenic combination immunotherapy of cT4a/bN+ stage GC (73). With continuous technological advancements, there is great potential for generating and publishing an even larger volume of such data in the future, thus providing a robust basis for the precise application of anti-angiogenic therapy in GC management.

## 6 Conclusion

Anti-angiogenic therapy has shown promise in advanced GC treatment but faces significant challenges including limited therapeutic efficacy, complex resistance mechanisms and an absence of reliable predictive markers. These challenges have prompted researchers to explore novel angiogenesis inhibitors, bispecific antibodies, and comprehensive predictive models. With enhanced understanding of tumor angiogenesis and the tumor microenvironment, combination therapies especially combining anti-angiogenic agents with immune checkpoint inhibitors have demonstrated remarkable synergistic effects. Several ongoing prospective clinical trials focusing on perioperative and first-line treatments may provide additional insights for the potential expansion of anti-angiogenic therapy beyond current second-line applications. Precision patient selection through validated biomarker approaches will be essential for advancing these therapeutic strategies. This requires developing prediction models that integrate multi-dimensional biomarker data to identify optimal candidates for earlier therapeutic intervention, thereby expanding the population of gastric cancer patients who can benefit from these targeted treatments beyond current indications.
